# A real‐world comparison of docetaxel versus abiraterone acetate for metastatic hormone‐sensitive prostate cancer

**DOI:** 10.1002/cam4.4184

**Published:** 2021-08-10

**Authors:** Igor Tsaur, Isabel Heidegger, Jasmin Bektic, Mona Kafka, Roderick C. N. van den Bergh, Jarmo C. B. Hunting, Anita Thomas, Maximilian P. Brandt, Thomas Höfner, Eliott Debedde, Constance Thibault, Paola Ermacora, Fabio Zattoni, Silvia Foti, Alexander Kretschmer, Guillaume Ploussard, Severin Rodler, Gunhild von Amsberg, Derya Tilki, Christian Surcel, Barak Rosenzweig, Moran Gadot, Giorgio Gandaglia, Robert Dotzauer, EAU‐YAU Prostate Cancer Working Party

**Affiliations:** ^1^ Department of Urology and Pediatric Urology University Medicine Mainz Mainz Germany; ^2^ Department of Urology Medical University Innsbruck Innsbruck Austria; ^3^ Department of Urology St Antonius Hospital Utrecht The Netherlands; ^4^ Department of Medical Oncology European Georges Pompidou Hospital Assistance Publique des Hôpitaux de Paris Paris Descartes University Paris France; ^5^ Unit of Urology Santa Maria della Misericordia Academic Medical Center Hospital Udine Italy; ^6^ Division of Oncology/Unit of Oncology Urological Research Institute IRCCS Ospedale San Raffaele Milan Italy; ^7^ Department of Urology Ludwig‐Maximilians‐University of Munich Munich Germany; ^8^ Department of Urology La Croix du Sud Hospital Toulouse France; ^9^ Institut Universitaire du Cancer Toulouse—Oncopole Toulouse France; ^10^ Department of Urology University Hospital Hamburg‐Eppendorf Hamburg Germany; ^11^ Martini‐Klinik Prostate Cancer Center University Hospital Hamburg‐Eppendorf Hamburg Germany; ^12^ Department of Urology University Hospital‐Hamburg Eppendorf Hamburg Germany; ^13^ Center of Urologic Surgery, Dialysis and Renal Transplantation Fundeni Clinical Institute Bucharest Romania; ^14^ Department of Urology Sheba Medical Center Tel Hashomer, affiliated with Sackler Faculty of Medicine Tel Aviv University Tel Aviv Israel; ^15^ Oncology Institute Sheba Medical Center Tel‐Hashomer Israel; ^16^ Division of Oncology/Unit of Urology Urological Research Institute IRCCS Ospedale San Raffaele Milan Italy

**Keywords:** chemotherapy, hormonal therapy, hormone‐sensitive, metastasis, prostate cancer

## Abstract

**Background:**

Docetaxel (D) or secondary hormonal therapy (SHT) each combined with androgen deprivation therapy (ADT) represent possible treatment options in males with metastasized hormone‐sensitive prostate cancer (mHSPC). Real‐world data comparing different protocols are lacking yet. Thus, our objective was to compare the efficacy and safety of abiraterone acetate (AA)+ADT versus D+ADT in mHSPC.

**Methods:**

In a retrospective multicenter analysis including males with mHSPC treated with either of the aforementioned protocols, overall survival (OS), progression‐free survival 1 (PFS1), and progression‐free survival 2 (PFS2) were assessed for both cohorts. Median time to event was tested by Kaplan–Meier method and log‐rank test. The Cox‐proportional hazards model was used for univariate and multivariate regression analyses.

**Results:**

Overall, 196 patients were included. The AA+ADT cohort had a longer PFS1 in the log‐rank testing (23 vs. 13 mos., *p* < 0.001), a longer PFS2 (48 vs. 33 mos., *p* = 0.006), and longer OS (80 vs. 61 mos., *p* = 0.040). In the multivariate analyses AA+ADT outperformed D+ADT in terms of PFS1 (HR = 0.34, 95% CI = 0.183–0.623; *p* = 0.001) and PFS2 (HR = 0.33 95% CI = 0.128–0.827; *p* = 0.018), respectively, while OS and toxicity rate were similar between both groups.

**Conclusions:**

AA+ADT is mainly associated with a similar efficacy and overall toxicity rate as D+ADT. Further prospective research is required for validation of the clinical value of the observed benefit of AA+ADT for progression‐free end‐points.

## Graphical abstract

Abiratreone acetate outperforms docetaxel in terms of PFS and PFS2, while the impact on OS as well as the rate of side effects is similar between both groups in real‐life utilization. Prospective randomized trials of available agents in mHSPC are required to generate high‐level evidence to facilitate sensible drug selection

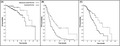

## 1 INTRODUCTION

The treatment pattern of metastasized hormone‐sensitive prostate cancer (mHSPC) evolved dramatically in the last 5 years. Since androgen deprivation therapy (ADT) remains the backbone of the systemic treatment of mHSPC, combining it either with docetaxel (D) or novel secondary hormonal therapy (SHT) yielded remarkable survival improvement and revolutionized standard care in the clinical routine.^1^ In this context, D was the first to achieve a significant reduction of the risk of death by 28% in the long‐term follow‐up of the CHAARTED trial.^2^ Due to conflicting results from the CHAARTED and STAMPEDE (arm C) trials, there is still no consensus if it should be used for males with low‐volume and/or recurrent disease.^2^, ^3^ Later, LATITUDE demonstrated a decrease in risk of death by 34% in the final assessment of overall survival (OS) using abiraterone acetate (AA) in men with a newly diagnosed high‐risk mHSPC.^4^ Results of STAMPEDE (arm G) confirmed survival advantage of AA in all‐comers leaving however, the label of AA being restricted to the LATITUDE inclusion criteria.^5^ Given this constraint, application of AA+ADT in low‐risk mHSPC requires individualized arrangement of the cost coverage by insurance company.

Most recently, ARCHES showed a decrease in risk of radiographic progression or death by 61% for adding enzalutamide (ENZ) to ADT.^6^ In ENZAMET, ENZ lowered the risk of death by 33% as compared to first‐generation antiandrogens.^7^ Apalutamide (APA) has recently been registered after decreasing the risk of death at 24 mos. by 33% as well as that of radiographic progression or death at 24 months by 61% in the TITAN trial.^8^

In the clinical practice, selection of the preferred agent in the personalized scenario of mHSPC is delicate given the lack of predictive tumor or patient characteristics, robust biomarkers and nomograms. Based on the longer period since registration, D and AA have been putatively the most commonly used drugs in mHSPC in Europe in the last years. Notably, contemporary network meta‐analysis by Marchioni and colleagues revealed no difference between D+ADT and AA+ADT in OS or toxicity, but favored AA+ADT in terms of disease progression rate.^9^ Since to our best knowledge real‐life data comparing both drugs has not been published yet, we aimed at assessing efficacy and safety of D+ADT versus AA+ADT in mHSPC patients treated at European and Israeli tertiary referral centers.

## 2 PATIENTS AND METHODS

### 2.1 Data collection

In this retrospective multicenter analysis including data of 11 institutions (Mainz, Innsbruck, Utrecht, Paris, Udine, Milan, Munich, Toulouse, Bucharest, Tel Aviv and Hamburg; Figure S1), clinical characteristics of males with mHSPC were collected into the database in accordance to the local ethical standards and the declaration of Helsinki. Inclusion criteria were receiving first‐line treatment with D (up to 6 cycles, standard dose 75mg/m2 body surface Q3W) or AA (1000 mg qd). Prednisone was voluntary for D and mandatory for AA. Long‐term ADT for localized disease was an exclusion criterion. D was initiated between 11/2012 and 02/2020 and AA between 05/2013 and 07/2020. Five patients underwent treatment with AA for mHSPC as off label use before its approval in 2018. Adverse events were classified according to the Common Terminology of Adverse Events of the National Cancer Center Version 5.^10^ Clinical outcomes were investigated by analysis of OS (time from start of ADT until death from any cause) and progression free‐survival 1/2 (PFS1/PFS2, time from start of ADT to clinical, biochemical or radiographic progression during first/second‐line or death from any cause).

### 2.2 Statistical analysis

Chi‐squared and Mann–Whitney *U* test were used for group comparison between treatment cohorts. Median time to event was tested by Kaplan–Meier method and log‐rank test. After univariate testing, multivariate survival analysis regression was performed with the Cox proportional‐hazards model with the variables (enter method). For the multivariate testing, variables were chosen with regard to their clinical relevance and to the number of events in the respective category. Significance level was set to *p* < 0.05. Statistical analysis was performed using IBM SPSS Statistics Version 20 (IBM Corp.).

## 3 RESULTS

### 3.1 Baseline characteristics

Clinical characteristics of 196 included patients are presented in Table 1. Median age at the start of first‐line therapy was 66 years with a median PSA at diagnosis of 170 ng/ml. Eighty percent of men had at least ISUP 4–5 grading, while 83.6% presented with a de‐novo mHSPC. Seventy percent of patients had a high‐volume disease according to CHAARTED criteria^11^ and 81.0% had a high‐risk disease according to LATITUDE criteria.^12^ Males from the D+ADT cohort were younger, had a better ECOG performance status, higher ISUP grading, higher median PSA at diagnosis and nodal metastatic burden.

**TABLE 1:** Clinical baseline characteristics and group comparison (abiraterone aetate vs. docetaxel first‐line therapy) of metastasized hormone‐sensitive prostate cancer patients

Variable	All patients (*n =* 196)		Abiraterone acetate (*n =* 48)		Docetaxel (*n =* 148)		*p* value
	%	*n*	%	*n*	%	*n*	
Median age (year). IQR	65.0	60.0–72.0	69.0	62.0–79.0	65.0	59.0–71.0	**0.001**
ECOG							
0	63.2	117	50.0	20	66.9	97	**0.047**
1	30.3	56	40.0	16	27.6	40	
2	6.5	12	10.0	4	5.5	8	
Median PSA at diagnosis (ng/ml). IQR	170	46.0–600.0	120.0	22.0–237.0	190.0	57.5–729.0	**0.005**
ISUP grade							
1	4.2	7	7.1	3	3.2	4	**0.047**
2	4.2	7	2.4	1	4.8	6	
3	11.4	19	11.9	5	11.3	14	
4	25.9	43	40.5	17	21.0	26	
5	54.2	90	38.1	16	59.7	74	
Primary tumor treatment							0.050
Radical prostatectomy	8.6	17	12.5	6	7.4	11	
Radiotherapy	7.6	15	22.9	11	2.7	4	
No primary treatment	83.6	164	64.5	31	89.8	133	
Disease volume (CHAARTED)							0.573
High	70.0	126	66.7	30	71.1	96	
Low	30.0	54	33.3	15	28.9	39	
Disease risk (LATITUDE)							1.000
High	81.0	115	81.8	36	80.6	79	
Low	19.0	54	18.2	8	19.4	19	
Nodal metastasis							**0.014**
N1	27.5	54	29.2	14	35.1	40	
M1a	39.3	77	39.6	19	50.9	58	
None	33.2	65	31.2	15	14.0	16	
Osseous metastasis							0.808
Axial skeletton	26.0	51	18.7	9	29.6	42	
Outside axial skeletton	3.0	6	2.1	1	3.5	5	
Both sites	52.5	103	56.2	27	53.5	76	
None	18.3	36	22.9	11	13.4	19	
Visceral metastasis							
Lung	12.2	24	18.7	9	13.6	15	0.696
Liver	3.6	7	2.1	1	5.4	6	
Brain	1.0	2			1.8	2	
Peritoneum	0.5	1	2.1	1			
Other	2.8	5	4.2	2	2.7	3	
None	80.0	157	72.9	35	76.4	84	
Number of docetaxel cycles first‐line							
4					3.1	4	
5					11.0	14	
6					85.8	109	
Second‐line treatment							
Docetaxel	16.0	23	69.6	16	5.8	7	
Cabazitaxel	11.8	17			14.2	17	
Abiraterone acetate	31.9	46			37.5	45	
Enzalutamide	31.2	45	21.7	5	33.3	40	
Radium223	6.2	9	8.7	2	5.8	7	
LuPSMA	0.7	1			0.8	1	
Other	2.1	3			2.5	3	
Third‐line treatment							
Docetaxel	12.3	9	18.0	2	11.5	7	
Cabazitaxel	31.5	23	9.0	1	36.1	22	
Abiraterone acetate	23.3	17			26.2	16	
Enzalutamide	23.3	17	63.6	7	16.4	10	
Radium223	2.7	2			3.3	2	
LuPSMA	1.4	1	9.0	1	3.3	2	
Other	5.5	4			3.0	2	
Fourth‐line treatment							
Docetaxel	13.8	4	40.0	2	8.3	2	
Cabazitaxel	31.0	9	40.0	2	29.2	7	
Abiraterone acetate	6.9	2			8.3	2	
Enzalutamide	24.1	7	20.0	1	25.0	6	
LuPSMA	13.8	4			16.7	4	
Other	10.3	3			12.5	3	

Abbreviations: ECOG, EASTERN cooperative oncology group; IQR, interquartile range; ISUP, international society of urological pathology; LuPSMA, lutetium‐177 prostate‐specific membrane antigen; PS, performance status; PSA, prostate‐specific antigen.

Bold: Statistically significant *p* values. For binary and ordinal variable percentage and number are indicated. For continuous variables median and interquartile range are indicated.

### 3.2 Survival analyses

**FIGURE 1:**
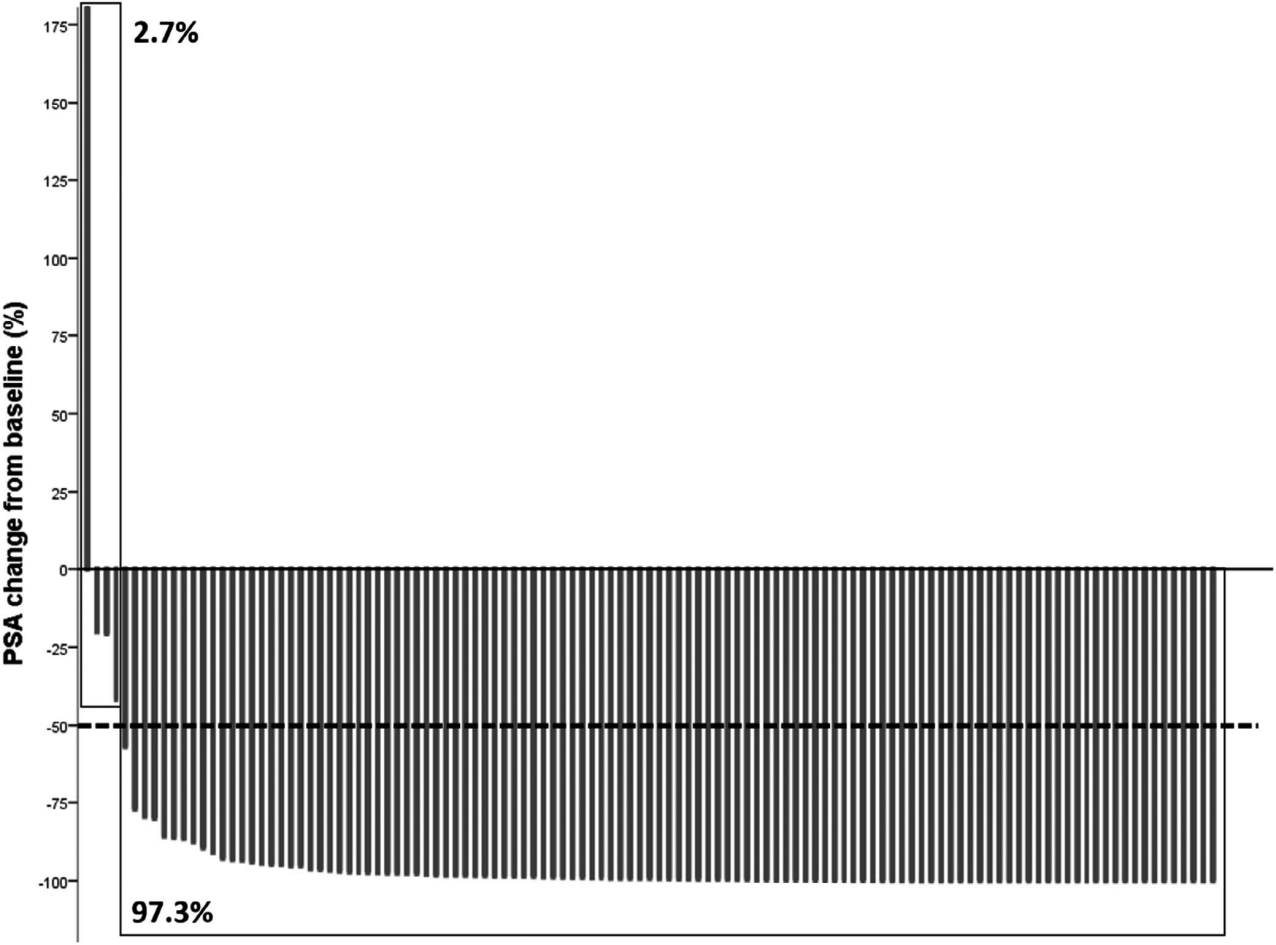
PSA change (%) from baseline to nadir in first‐line therapy for all patients. PSA, prostate‐specific antigen

**FIGURE 2:**
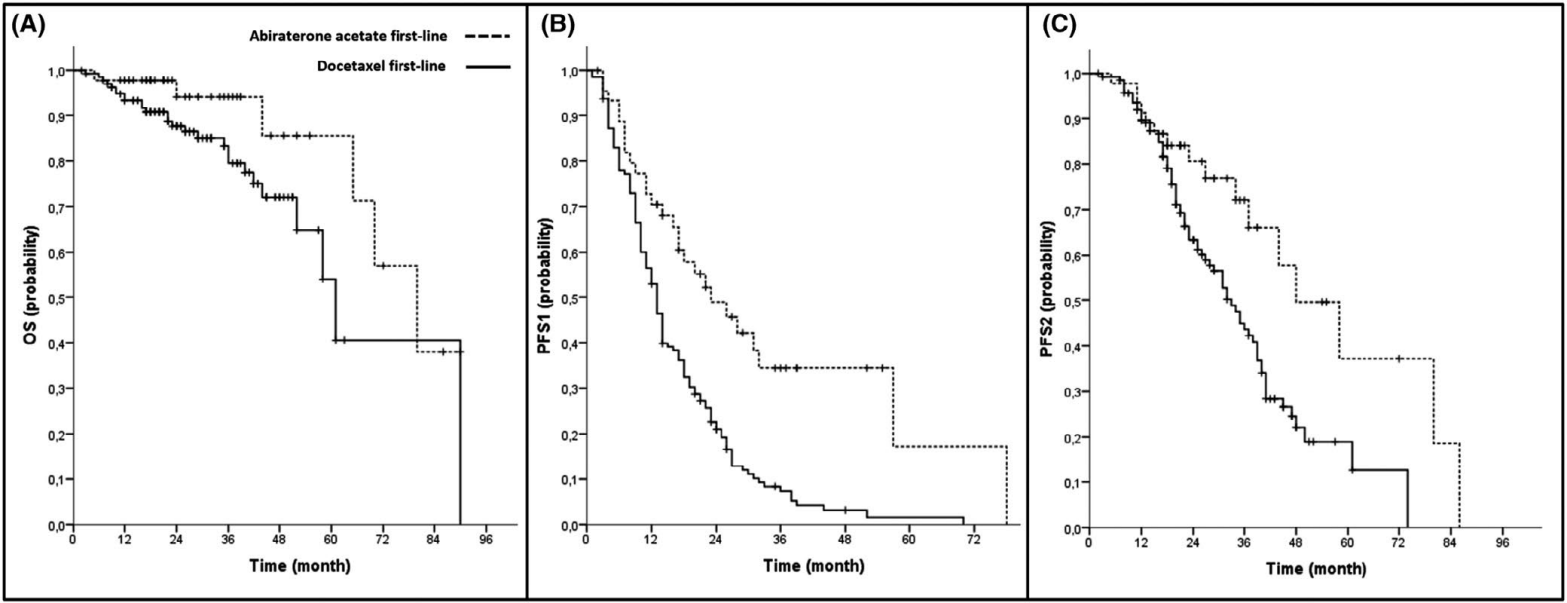
Kaplan–Meier curves for (A) overall survival, (B) progression‐free survival 1 (PFS1) and (C) progression‐free survival 2 (PFS2) for prostate cancer patients undergoing first‐line therapy with abiraterone acetate or docetaxel

Clinical outcomes of all patients and the respective cohort are presented in Table 2. During first‐line treatment, a mean PSA change to nadir of –88.96% (SD 89.32) could be noticed and 97.2% of all patients had a PSA response of more than 50% (PSA50; Figure 1). For all patients, median follow‐up time was 27 months, median PFS1 was 14 months, median PFS2 was 36 months and median OS was 70 months The AA+ADT cohort had a longer PFS1 in the log‐rank testing (23 vs. 13 mos., *p* < 0.001), a longer PFS2 (48 vs. 33 mos., *p* = 0.006), and longer OS (80 vs. 61 mos., *p* = 0.040). For Kaplan–Meier curves see Figure 2. At the time of data evaluation, 28 patients succumbed. The median follow‐up for survivors was 27 months. The overall rate of toxicity did not differ between both cohorts. In multivariate Cox regression analyses, AA+ADT outperformed D+ADT in regard to PFS1 (HR = 0.34, 95% CI = 0.183–0.623; *p* = 0.001) and PFS2 (HR = 0.33 95% CI = 0.128–0.827; *p* = 0.018). The results of the multivariate analyses did not change when the covariate “nodal metastasis” was replaced by “disease volume”. Results of the multivariate analysis are presented in Table 3 as well as those of uni‐ and further multivariate analyses in Tables [Link], [Link], [Link].

**TABLE 2:** Clinical outcome characteristics and group comparison (abiraterone acetate vs. docetaxel first‐line therapy) of metastasized hormone sensitive prostate cancer patients

Variable	All patients			Abiraterone acetate			Docetaxel			
	Mean	Median	95% CI	Mean	Median	95% CI	Mean	Median	95% CI	*p* value
FU time	29.71	27	18.000–40.000	32.44	26	18.000–39.000	29.13	27	18.000–41.000	0.757
OS	64.40	70	57.253–82.747	73.42	80	59.969–100.031	62.24	61	50.226–71774	**0.040**
PFS1	19.90	14	11.642–16.358	33.66	23	13.388–32.612	16.34	13	11.506–14.494	**<0.001**
PFS2	39.89	36	31.611–40.389	53.26	48	31.637–64.363	35.81	33	27.909–38.091	**0.006**
PSA change from baseline to nadir first‐line mean, SD (%)	−88.96	89.32		−95.83	11.09		−86.83	102.03		0.155
PSA change from baseline to start of second‐line mean, SD (%)	−98.63	120.09		−90.54	30.44		−98.85	133.88		**0.015**
PSA50 response first‐line										
Yes (%, *n*)	97.2	140		97.0	33		97.3	107		0.947
No (%, *n*)	2.7	4		3.0	1		2.7	3		
CTCAE in first‐line										
Grade I–II (%, *n*)	86.30	138		87.90	29		85.80	109		0.760
Grade III–IV (%, *n*)	13.80	22		12.10	4		14.20	18		

Abbreviations: 95% CI, 95% confidence interval; CTCAE, common terminology criteria for adverse events; FU time, follow up time; OS, overall survival; PFS1/PFS2, progression‐free survival 1/2; PSA, prostate‐specific antigen; PSA50 response, PSA decrease of ≥50% from baseline; SD, standard deviation.

Bold: Statistically significant *p* values.

**TABLE 3:** Multivariate Cox regression analyses of clinical outcomes (OS, PFS1 and PFS2) and patient characteristics

Risk factors for occurrence of event(s)	End points								
	OS			PFS1			PFS2		
	HR	95% CI	*p* value	HR	95% CI	*p* value	HR	95% CI	*p* value
First‐line treatment									
Abiraterone acetate	0.14	0.017–1.128	0.065	0.34	0.183–0.623	**0.001**	0.33	0.128–0.827	**0.018**
Docetaxel	1.0 (ref.)			1.0 (ref.)			1.0 (ref.)		
Nodal metastasis									
Yes	3.45	0.436–27.251	0.240	1.24	0.644–2.375	0.522	0.98	0.427–2.248	0.960
No	1.0 (ref.)			1.0 (ref.)			1.0 (ref.)		
ISUP grading	1.05	0.639–1.716	0.856	1.09	0.882–1.348	0.425	1.00	0.758–1.327	0.985
PSA at diagnosis	1.00	0.999–1.001	0.368	1.00	1.000–1.000	0.867	1.00	1.000–1.000	0.483
Age	0.99	0.937–1.038	0.592	0.98	0.960–1.007	0.157	1.01	0.982–1.046	0.396

Abbreviations: CI, confidence interval; HR, hazard ratio; ISUP grading, international society of urological pathology grading; OS, overall survival; PFS1/PFS2, progression‐free survival 1/2; Ref., reference.

Bold: Statistically significant *p* values.

## 4 DISCUSSION

Docetaxel or SHT each combined with ADT represent the standard of care in males with mHSPC. Since no data exist in favor of any strategy from prospective trials comparing one of these concepts with the other in a direct randomized fashion, multiplied by the shortage of established predictive factors, uncertainty about choosing the most promising treatment option in a specific individual remains. As a result, shared decision‐making relies on second‐order sources of evidence, existing disorders constituting risk factors for related adverse events as well as patient preference. In this setting, comparison of a real‐life efficacy and safety of the available agents may provide findings with a direct practical orientation complementing results generated by meta‐analysis of clinical trials which command the current field of evidence.

Most recently, Chen and collaborators presented a meta‐analysis including 10 trials and 16 publications comparing various systemic combination therapies and generated the surface under the cumulative ranking curve (SUCRA) for treatment ranking.^13^ By doing so, SUCRA (standing for the probability to be the best treatment) revealed that AA+ADT and ENZ+ADT outperformed other protocols in terms of both OS (80% and 79%, respectively) and failure‐free survival (FFS; 92% and 93%, respectively). This is in line with the outcomes of the network meta‐analysis by Tan and collaborators including seven trials.^14^ Herein, AA+ADT suggested an improved OS with 97% certainty for a 19% reduction in risk of death as well as a more favorable FFS with 100% certainty for a 50% decrease in risk of progression or death compared to D+ADT. Another network meta‐analysis by Feyerabend et al. investigated relative benefits of AA+ADT or D+ADT on OS, radiographic progression‐free survival (rPFS) and quality of life (QoL) measured by the Brief Pain Inventory, and the Functional Assessment of Cancer Therapy‐Prostate questionnaire using patient outcomes from CHAARTED, STAMPEDE, LATITUDE, and GETUG‐AFU 15 (negative for OS benefit of D+ADT over ADT alone) trials.^15^ They found that AA+ADT was at least as effective as D+ADT in reducing the risk of death and outperformed it at preventing disease progression and improving QoL for at least 1 year of treatment. Similarly, a recent systematic review and network meta‐analysis of seven trials by Sathianathen and coworkers comparing D+ADT, AA+ADT, ENZ+ADT, and APA+ADT found no difference in OS.^16^ It is noteworthy that AA and ENZ were comparable to each other and preferred over both D and APA in terms of PFS. Recently, Sydes et al. comparatively assessed contemporaneously randomized men to D+ADT (*n* = 189) or AA+ADT (*n* = 377) in different arms of STAMPEDE.^17^ It is important to keep in mind that STAMPEDE was in fact not designed to test for differences in outcomes between these two arms. However, this assessment undoubtedly yielded the most robust data for comparison of these both treatment strategies thus far. While no evidence in favor of any protocol has been observed in relation to OS, cancer‐specific or metastatic progression‐free survival, number of symptomatic skeletal events or toxicity, the risk for both FFS and PFS was diminished by AA+ADT by 49% and 35%, respectively. Importantly, our PFS definition corresponds to that of FFS in STAMPEDE in contrast to their PFS which excludes biochemical progression. Thus, even ruling out the events of PSA rise from consideration as progression, Sydes and collaborators demonstrated a benefit for AA+ADT over D+ADT underpinning its robustness. In accordance with this data as well as those of the most meta‐analysis, our assessment yielded no significant difference between both treatment arms in relation to OS but in terms of PFS. Thus, PFS1 was prolonged by 60% in the AA+ADT cohort of our study corroborating the aforementioned results. Clinical value of this advantage in PFS should be interpreted with caution. Delay of progression has been demonstrated to predict survival in PCa. Thus, metastasis‐free survival (MFS) has been shown to be a strong surrogate of OS in localized PCa in a meta‐analysis by Xie and colleagues.^18^ The same holds true for nonmetastatic castration‐resistant PCa (CRPC), as a strong association of MFS with OS was revealed in a retrospective assessment of 1207 men from the SPARTAN trial by Smith et al.^19^ In metastatic CRPC, rPFS was proposed to serve as a potential surrogate endpoint of OS in the meta‐analysis by Chen and coauthors.^20^ In mHSPC, Martini et al. identified progression within 6 months as the best surrogate for OS relying on data of 790 patients from the CHAARTED trial.^21^ Notwithstanding this correlation, the lack of OS difference in our real‐world analysis might be attributable to the factual flimsy difference of the impact of AA+ADT or D+ADT on OS, which might be only unmasked in a sufficiently powered prospective trial. Moreover, a relatively short follow‐up of the current study with a limited number of deaths precludes its robust estimation.

Theoretically, relationship between PFS and OS may be considerably influenced by subsequent therapies, particularly by the second‐line treatment. The impact of subsequent treatments may hide the effect of the first‐line treatment such that OS benefits are not always observed despite significant improvements in PFS.^22^ Also imaginable is the scenario in which first‐line therapies may have a short PFS but extend OS by sensitizing tumor cells to second‐line agents.^23^ In the current investigation, 69.6% of progressing males received D in the AA+ADT group as compared to 70.8% of those from the D+ADT group who received SHT upon progression. Taken together, a similar rate of patients who underwent second‐line treatment received a sequence D‐SHT or vice‐versa in both cohorts. Interestingly, we could demonstrate an advantage of AA+ADT utilized in the first‐line setting over D+ADT on the PFS2 as well, whereby it decreased the risk of 2^nd^ disease progression or death by 67%. This endpoint is currently gaining increasing attention in clinical trials. It has been speculated that it could be a more precise surrogate of the benefit of sequence therapy than PFS.^24^ In a contemporary meta‐analysis of 15 studies on different tumor entities including PCa, Chowdhury and colleagues demonstrated that PFS2 strongly correlated with OS proposing its usage before OS data get mature or if OS cannot be assessed.^22^ Using the data of the PROREPAIR‐B study which investigated the impact of germline DNA repair mutations on the outcomes of patients with mCRPC,^25^ Lorente and coauthors observed that PFS2 outperformed PFS1 as a predictor of OS. Since the number of events during the second‐line treatment in our analysis was low precluding far‐reaching conclusions, further research is warranted to shed light on the influence of both AA+ADT and D+ADT on PFS2. Particularly in trials in which the choice of the agents in the second‐line indication is not mandated by the study protocol, PFS2 might compensate for this possible bias as compared to PFS1.

Another important aspect to consider is whether prolonged PFS translates into improved health‐related quality of life (HRQoL). Interestingly, findings in lung and breast cancer demonstrated that cancer progression was associated with a statistically significant worsening in HRQoL supporting the value of PFS as a patient‐relevant end point and emphasizing importance of avoiding progression and prolonging PFS to maintain HRQoL.^26^ Moreover, Beer and coauthors reported on a predictive value of several HRQoL domains on radiographic PFS in PREVAIL and AFFIRM studies which investigated the effects of ENZ in pre‐ and post‐chemotherapy setting of mCRPC, respectively.^27^ Notably, these domains differed in part between both studies probably reflecting the impact of the different treatment line and/or influence of the previous cytotoxic therapy on their value. On the other hand, alleviation of symptoms that patients gain from treatment mediating tumor regression or stabilization must be weighted against the toxic effects provoked by agents themselves.^28^ Indeed, a recent systematic review and quantitative analysis of 38 clinical trials of tumor patients including two PCa studies by Kovic et al. did not prove association between PFS and HRQoL challenging the assumption that interventions prolonging PFS also improve HRQoL.^29^ In line with these findings, Hwang and collaborators questioned the validity of PFS as a surrogate for HRQoL.^30^ In their assessment of 147 Phase three trials reporting HRQoL outcomes, only a weak correlation between PFS and improvement in HRQoL could be observed. All‐in‐all, association of PFS with HRQoL appears to depend inter alia on the cancer entity, tumor stage and treatment line as well as toxicity of the treatment. In our study, no difference could be observed between both therapies in terms of the rate of side effects. Undoubtedly, further research is warranted in order to illuminate the relationship between PFS and HRQoL in mHSPC.

Our analysis has some limitations to be taken into account. First, it is a retrospective assessment with all inherent flaws and possible biases. Second, the study is based on a limited sample size predominantly in relation to the AA+ADT cohort. Third, due to the low sample size we could not shed light on association of different types of progression with survival end‐points evaluating a composite progression. Fourth, due to the multicentre study design and the varying number of enrolled patients per site, heterogeneity in treatment‐related proceedings and data processing cannot be avoided. Fifth, there are other factors like the influence of comorbidities, blood levels as well as insurance status that are not examined by this study, which could have influenced the choice of agent. Notwithstanding these drawbacks, we believe to have contributed to the current understanding of mHSPC treatment by providing the first real‐world data comparing AA+ADT versus D+ADT. We could demonstrate that AA+ADT is a better option than D+ADT with regards to PFS and PFS2, while the impact on OS as well as the rate of side effects was similar between both groups. Further research is warranted to clarify clinical significance of the observed advantage and if it can justify declaration of AA+ADT as the primary treatment option out of these two. Hitherto, preexisting comorbidities, local drug availability, label/cost coverage issues and patient preferences should be part of the shared decision‐making between D+ADT and SHT+ADT.

## 5 CONCLUSIONS

AA+ADT outperforms D+ADT in terms of PFS and PFS2, while the impact on OS as well as the rate of side effects is similar between both groups in real‐life utilization. Prospective randomized trials of available agents in mHSPC are required to generate high‐level evidence to facilitate sensible drug selection.

## CONFLICT OF INTEREST

The authors declare that there are no conflicts of interest.

## ETHICAL APPROVAL STATEMENT

This study was performed in accordance to the local ethical standards and the declaration of Helsinki.

## Supporting information

Fig S1

Table S1

Table S2

Table S3

## Data Availability

The data that supports the findings of this study are available in the supplementary material of this article.
