# Supplementary material for: A real‐world comparison of docetaxel versus abiraterone acetate for metastatic hormone‐sensitive prostate cancer
Source: Cancer Med. 2021 Aug 10;10(18):6354–64. doi: 10.1002/cam4.4184 (PMC8446402; doi:10.1002/cam4.4184)
Supplement: Supplementary file 1 — Fig S1 [file CAM4-10-6354-s004.pdf]

Docetaxel		Abiraterone acetate	
36 Utrecht	36 Paris	17 Tel Aviv	12 Mainz
22 Udine	16 Innsbruck	7 Bukarest	6 Paris
15 Munich	15 Mainz	4 Innsbruck	2 Munich
5 Toulouse	5 Hamburg		
