# Supplementary material for: A real‐world comparison of docetaxel versus abiraterone acetate for metastatic hormone‐sensitive prostate cancer
Source: Cancer Med. 2021 Aug 10;10(18):6354–64. doi: 10.1002/cam4.4184 (PMC8446402; doi:10.1002/cam4.4184)
Supplement: Supplementary file 2 — Table S1 [file CAM4-10-6354-s002.docx]

Supplementary table 1 – Univariate Cox regression analyses of clinical outcome (OS, PFS1 and PFS2) and patient characteristics.

| Risk factors for occurence of event(s) |  | End points | | | | | | | | | | |
| --- | --- | --- | --- | --- | --- | --- | --- | --- | --- | --- | --- | --- |
|  |  | OS | | |  | PFS1 | | |  | PFS2 | | |
|  |  | HR | 95% CI | p value |  | HR | 95% CI | p value |  | HR | 95% CI | p value |
| First-line treatment |  |  |  |  |  |  |  |  |  |  |  |  |
| Abiraterone acetate |  | 0.052 | 0.003 - 0.896 | **0.042** |  | 1.099 | 0.378 - 3.193 | 0.863 |  | 0.367 | 0.123 - 1.092 | 0.072 |
| Docetaxel |  | 1.0 (Ref.) |  |  |  | 1.0 (Ref.) |  |  |  | 1.0 (Ref.) |  |  |
| Osseous metastases |  |  |  |  |  |  |  |  |  |  |  |  |
| yes |  | 0.060 |  | 0.377 |  | 1.602 | 0.455 - 5.635 | 0.463 |  | 0.756 | 0.132 - 4.314 | 0.753 |
| no |  | 1.0 (Ref.) |  |  |  | 1.0 (Ref.) |  |  |  | 1.0 (Ref.) |  |  |
| Nodal metastases |  |  |  |  |  |  |  |  |  |  |  |  |
| yes |  | 1636288.281 |  | 0.980 |  | 0.976 | 0.429 - 2.218 | 0.954 |  | 0.941 | 0.328 - 2.698 | 0.910 |
| no |  | 1.0 (Ref.) |  |  |  | 1.0 (Ref.) |  |  |  | 1.0 (Ref.) |  |  |
| Visceral metastases |  |  |  |  |  |  |  |  |  |  |  |  |
| yes |  | 2.465 | 0.537 - 11.323 | 0.246 |  | 2.208 | 1.011 - 4.822 | **0.047** |  | 1.585 | 0.628 - 3.999 | 0.329 |
| no |  | 1.0 (Ref.) |  |  |  | 1.0 (Ref.) |  |  |  | 1.0 (Ref.) |  |  |
| Disease volume (CHAARTED) |  |  |  |  |  |  |  |  |  |  |  |  |
| high |  | 49.299 | 0.115 - 21078 | 0.207 |  | 1.370 | 0.573 - 3.278 | 0.479 |  | 2.969 | 0.750 - 11.754 | 0.121 |
| low |  | 1.0 (Ref.) |  |  |  | 1.0 (Ref.) |  |  |  | 1.0 (Ref.) |  |  |
| Liver metastases |  |  |  |  |  |  |  |  |  |  |  |  |
| yes |  | 0.850 |  | 0.999 |  | 0.619 | 0.054 - 7.129 | 0.701 |  | 5.752 | 0.334 - 99.154 | 0.228 |
| no |  | 1.0 (Ref.) |  |  |  | 1.0 (Ref.) |  |  |  | 1.0 (Ref.) |  |  |
| De novo disease |  |  |  |  |  |  |  |  |  |  |  |  |
| yes |  | 1.120 | 0.361 - 3.477 | 0.844 |  | 0.778 | 0.360 - 1.683 | 0.524 |  | 0.870 | 0.348 - 2.175 | 0.766 |
| no |  | 1.0 (Ref.) |  |  |  | 1.0 (Ref.) |  |  |  | 1.0 (Ref.) |  |  |
| Age |  | 1.003 | 0.941 - 1.069 | 0.921 |  | 0.966 | 0.941 - 0.993 | **0.012** |  | 1.041 | 1 .000 - 1.084 | 0.050 |
| ISUP grading |  | 1.065 | 0.572 - 1.983 | 0.843 |  | 1.093 | 0.874 - 1.368 | 0.434 |  | 1.058 | 0.776 - 1.443 | 0.721 |
| PSA at diagnosis |  | 1.000 | 0.999 - 1.000 | 0.622 |  | 1.000 | 1.000 - 1.000 | 0.825 |  | 1.000 | 1.000 - 1.000 | 0.169 |
| PSA change from baseline to nadir first line |  | 0.981 | 0.895 - 1.075 | 0.680 |  | 1.028 | 0.989 - 1.068 | 0.161 |  | 0.975 | 0.927 - 1.026 | 0.334 |
| PSA change from baseline to end of first-line |  | 1.038 | 0.998 - 1.08 | 0.066 |  | 0.979 | 0.957 - 1.002 | 0.079 |  | 1.030 | 1.005 - 1.056 | **0.019** |
| ECOG PS |  | 1.741 | 0.595 - 5.095 | 0.311 |  | 0.998 | 0.617 - 1.615 | 0.994 |  | 2.237 | 1.215 - 4.118 | **0.010** |
| Year of starting treatment |  | 1.649 | 0.762 - 3.569 | 0.204 |  | 1.224 | 0.903 - 1.659 | 0.192 |  | 1.719 | 1.076 - 2.746 | **0.024** |
| CI = confidence interval; HR = hazard ratio; OS = overall survival; PFS1/PFS2 = progression-free survival 1/2; Ref. = reference; ISUP grading = International Society of Urological Pathology grading. ECOG = Eastern Cooperative Oncology Group; PS = performance status | | | | | | | | | | | | |
| Bold: statistically significant p values. | | | | | | | | | | | | |
