# Supplementary material for: A real‐world comparison of docetaxel versus abiraterone acetate for metastatic hormone‐sensitive prostate cancer
Source: Cancer Med. 2021 Aug 10;10(18):6354–64. doi: 10.1002/cam4.4184 (PMC8446402; doi:10.1002/cam4.4184)
Supplement: Supplementary file 3 — Table S2 [file CAM4-10-6354-s003.docx]

Supplementary table 2 - Multivariate Cox regression analyses of clinical outcomes (OS, PFS1 and PFS2) and patient characteristics.

| Risk factors for occurence of event(s) |  | End points | | | | | | | | | | |
| --- | --- | --- | --- | --- | --- | --- | --- | --- | --- | --- | --- | --- |
|  |  | OS | | |  | PFS1 | | |  | PFS2 | | |
|  |  | HR | 95% CI | p value |  | HR | 95% CI | p value |  | HR | 95% CI | p value |
| First-line treatment |  |  |  |  |  |  |  |  |  |  |  |  |
| Abiraterone acetate |  | 0.13 | 0.016 - 1.056 | 0.056 |  | 0.38 | 0.383 - 0.692 | **0.001** |  | 0.25 | 0.109 - 0.566 | **0.001** |
| Docetaxel |  | 1.0 (ref.) |  |  |  | 1.0 (ref.) |  |  |  | 1.0 (ref.) |  |  |
| Disease volume (CHAARTED) |  |  |  |  |  |  |  |  |  |  |  |  |
| high |  | 1.61 | 0.578 - 4.490 | 0.361 |  | 1.13 | 0.714 - 1.781 | 0.608 |  | 1.31 | 0.770 - 2.221 | 0.320 |
| low |  | 1.0 (ref.) |  |  |  | 1.0 (ref.) |  |  |  | 1.0 (ref.) |  |  |
| ISUP grading |  | 1.14 | 0.692 - 1.867 | 0.613 |  | 1.02 | 0.838 - 1.230 | 0.876 |  | 0.89 | 0.701 - 1.123 | 0.887 |
| PSA at diagnosis |  | 1.00 | 0.998 - 1.000 | 0.158 |  | 1.00 | 1.000 - 1.000 | 0.973 |  | 1.00 | 0.999- 1.000 | 0.477 |
| Age |  | 0.99 | 0.944 - 1.038 | 0.669 |  | 0.98 | 0.956 - 1.003 | 0.090 |  | 1.01 | 0.982 - 1.034 | 0.574 |
| CI = confidence interval; HR = hazard ratio; OS = overall survival; PFS1/PFS2 = progression-free survival 1/2; Ref. = reference; ISUP grading = International Society of Urological Pathology grading. | | | | | | | | | | | | |
| Bold: statistically significant p values. | | | | | | | | | | | | |
