# Supplementary material for: A real‐world comparison of docetaxel versus abiraterone acetate for metastatic hormone‐sensitive prostate cancer
Source: Cancer Med. 2021 Aug 10;10(18):6354–64. doi: 10.1002/cam4.4184 (PMC8446402; doi:10.1002/cam4.4184)
Supplement: Supplementary file 4 — Table S3 [file CAM4-10-6354-s001.docx]

Supplementary table 3 - Multivariate Cox regression analyses of PFS2 and patient characteristics.

| Risk factors for occurence of event(s) |  | End point | | |
| --- | --- | --- | --- | --- |
|  |  | PFS2 | | |
|  |  | HR | 95% CI | p value |
| First-line treatment |  |  |  |  |
| Abiraterone acetate |  | 0.25 | 0.096 - 0.672 | **0.006** |
| Docetaxel |  | 1.0 (ref.) |  |  |
| Nodal metastasis |  |  |  |  |
| yes |  | 0.71 | 0.337 - 1.474 | 0.335 |
| no |  | 1.0 (ref.) |  |  |
| Second-line treatment |  |  |  |  |
| No chemotherapy |  | 0.51 | 0.258 - 1.004 | 0.051 |
| Chemotherapy |  | 1.0 (ref.) |  |  |
| ISUP grading |  | 0.98 | 0.757 - 1.273 | 0.887 |
| PSA at diagnosis |  | 1.00 | 0.999 - 1.001 | 0.780 |
| Age |  | 1.02 | 0.992 - 1.049 | 0.155 |
| CI = confidence interval; HR = hazard ratio; OS = overall survival; PFS1/PFS2 = progression-free survival 1/2; Ref. = reference; ISUP grading = International Society of Urological Pathology grading. | | | | |
| Bold: statistically significant p values. | | | | |
